# Efficacy of a Bispecific Antibody Co-Targeting VEGFA and Ang-2 in Combination with Chemotherapy in a Chemoresistant Colorectal Carcinoma Xenograft Model

**DOI:** 10.3390/molecules24162865

**Published:** 2019-08-07

**Authors:** Thomas Mueller, Juana Freystein, Henrike Lucas, Hans-Joachim Schmoll

**Affiliations:** 1 University Clinic for Internal Medicine IV, Hematology/Oncology, Medical Faculty of Martin Luther University Halle-Wittenberg, 06120 Halle (Saale), Germany; 2 Institute of Pharmacy, Martin Luther University Halle-Wittenberg, 06120 Halle (Saale), Germany; 3 University Hospital Halle (Saale), Martin Luther University Halle-Wittenberg, 06120 Halle (Saale), Germany

**Keywords:** anti-angiogenic therapy, VEGF, ANGPT2, Ang-2, bispecific antibody, CrossMab, bevacizumab, vanucizumab, colorectal cancer

## Abstract

Vascular endothelial growth factor (VEGF) inhibition by the addition of bevacizumab to the chemotherapy regimen of metastatic colorectal cancer leads to an improved outcome. However, anti-angiogenic tumor therapy targeting a single factor may be limited by complementary mechanisms. Angiopoietin-2 (Ang-2, ANGPT2) is another important factor that cooperates with VEGF to drive tumor angiogenesis. It was shown that high Ang-2 levels are associated with a poor clinical outcome of colorectal cancer patients treated with bevacizumab-containing therapy. Therefore, combined inhibition of VEGF and Ang-2 was supposed to improve anti-angiogenic therapy. Here, we evaluated the efficacy of a bispecific antibody (CrossMab) co-targeting VEGF and Ang-2 in combination with chemotherapy in a chemoresistant colorectal carcinoma model. Antitumor activity was evaluated in athymic nude mice bearing subcutaneous DLD1 xenograft tumors and treated with anti-VEGF (B20), anti-Ang-2 (LC06) and anti-VEGF/Ang-2 (CrossMab) antibodies. Chemotherapy consisted of 5-FU and irinotecan. Resected tumors were analyzed immunohistochemically. First, an impact of targeting each single factor but also a clear advantage of co-targeting both factors could be demonstrated. Accordingly, tumor tissue showed strong staining for VEGF and Ang-2. Chemotherapy alone was less effective. Efficient tumor growth inhibition could be achieved by treatment with anti-VEGF/chemotherapy, single CrossMab and CrossMab/chemotherapy, which resulted in 3 out of 10, 6 out of 10 and 10 out of 10 complete responses, respectively, during seven weeks. Complete retarded tumors were characterized by massive intratumoral necrosis surrounded by layers of vital tumor cells and connective tissue with CD31-positive vessels at the periphery. In some cases, a distinct feature known as vessel co-option could be observed. In conclusion, the data from this model clearly support the strategy of co-targeting VEGF and Ang-2 and further demonstrate the beneficial impact of co-treatment with chemotherapy. The clear superiority of the CrossMab-containing regimen compared to clinical standard anti-VEGF/chemotherapy warrants further analyses in other models.

## 1. Introduction

Tumor angiogenesis is a hallmark of cancer [1] and targeting angiogenesis represents an attractive therapeutic approach to treat cancer [2,3]. Vascular endothelial growth factor (VEGFA) is a key molecule in this context and plays a major role for angiogenesis, vascular permeability and tumor progression. The monoclonal antibody bevacizumab, which binds VEGFA, is one among several other angiogenesis inhibitors that are clinically approved. The addition of bevacizumab to chemotherapy has been shown to prolong patient survival in various cancers including colorectal cancer compared with chemotherapy alone [4]. However, intrinsic or developing resistance is frequently observed. This is due to the presence of overlapping and compensatory alternative angiogenic pathways providing escape mechanisms that limit the potential of VEGF-targeted therapies [5,6]. 

One escape mechanism may be mediated by angiopoietins, the functional ligands of the Tie2 receptor tyrosine kinase, which are involved in the remodeling of tumor vasculature [7]. Angiopoietin-1 (Ang-1, ANGPT1) acts as a regulator of vessel stabilization and maturation, whereas, activity of angiopoietin-2 (Ang-2, ANGPT2) induces angiogenic sprouting and leads to increased vessel plasticity. Tumors are typically characterized by a shift in the Ang-1/Ang-2 ratio toward Ang-2, which represents a proangiogenic switch [8]. In addition, increased expression or high serum levels of Ang-2 are associated with a poor prognosis [9,10,11]. Thus, Ang-2 is another important factor that cooperates with or partly compensates VEGF to drive tumor angiogenesis. In accordance with this notion high Ang-2 serum levels were associated with poor response and poor clinical outcome in patients with metastatic colorectal cancer treated with bevacizumab-containing therapy [12,13]. Therefore, combined inhibition of VEGF and Ang-2 had been supposed to improve anti-angiogenic therapy. 

Vanucizumab is a novel bispecific monoclonal antibody that has been recently created using CrossMAb technology and that binds both Ang-2 and VEGFA with high affinity [14,15]. In preclinical tests, vanucizumab inhibited angiogenesis, tumor growth, and micrometastatic seeds more effectively than mono-specific anti-Ang-2 or anti-VEGF mAbs, and led to an enhanced vessel maturation phenotype [16]. Recently, a phase I study evaluated the safety, pharmacokinetics, pharmacodynamics, and antitumor activity of single agent vanucizumab in patients with advanced solid tumors refractory to standard therapies [17]. Vanucizumab showed an acceptable safety and tolerability profile and modulated its angiogenic targets. Interestingly, encouraging antitumor activity was observed in one patient with renal cell cancer and one patient with metastatic colorectal cancer [17].

In the present study, we evaluated the efficacy of co-targeting VEGFA and Ang-2 using a bispecific antibody (CrossMab) in combination with chemotherapy compared with standard anti-VEGF/chemotherapy combination regimen in a chemoresistant colorectal carcinoma xenograft model thereby addressing two major problems in therapy of colorectal cancer: chemotherapy resistance and limited response to bevacizumab. 

## 2. Results

### 2.1. Impact of VEGF and Ang-2 single Targeting Compared to Co-targeting in the Colorectal Carcinoma Model

**Figure 1:**
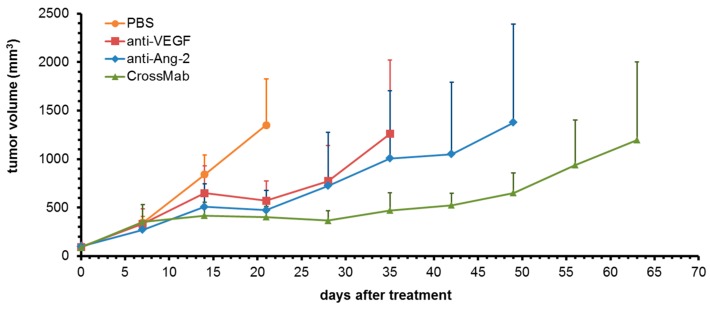
Analysis of tumor growth inhibition comparing single- vs. co-targeting VEGF and Ang-2. Mice were treated once weekly starting on day 0. Values are means of tumor volumes ± standard deviation (*n* = 3).

A first preliminary study using three mice per group was performed to analyze the impact of single targeting VEGF and Ang-2 using specific antibodies compared to co-targeting mediated by the bispecific CrossMab in the DLD1 colorectal carcinoma xenograft model (Figure 1). Each group consisted of similar mean tumor volumes at the start of treatment with 90 mm^3^, 94 mm^3^, 98 mm^3^ and 88 mm^3^ for PBS control, anti-VEGF, anti-Ang-2 and CrossMab, respectively. On day 21, after the start of treatment, the control group reached a mean tumor volume of 1350 mm^3^ with 1 out of 3 tumors exceeded 1500 mm^3^ and treatment was completed. All three antibodies induced a specific tumor response after a distinct lag phase of several days. They inhibited tumor growth and the treatments were continued until mean tumor volumes reached approximately that of the control group. Anti-VEGF treatment was discontinued on d35 when 1 tumor exceeded and 1 tumor had reached 1500 mm^3^. The other tumor was more retarded and had reached 400 mm^3^ at this time point. Within the anti-Ang-2 group 1 tumor showed complete retardation with tumor stasis during the course of treatment. On d49 the other 2 tumors had exceeded 1500 mm^3^ and treatment was completed. Overall, single targeting of VEGF and Ang-2 resulted in a similar response, which was characterized by an extended lag phase compared to the CrossMab group and a transient growth retardation phase after d14. The bispecific CrossMab most effectively inhibited tumor growth. One tumor showed complete retardation with tumor stasis until completion of the study on d63 when another tumor exceeded 1500 mm^3^. The third tumor had reached 1150 mm^3^ at this time point. In summary, these data confirmed Ang-2, in addition to VEGF, as another important target in anti-angiogenic therapy in this colorectal cancer model and demonstrated the clear advantage of co-targeting both factors. 

### 2.2. Impact of Co-targeting VEGF and Ang-2 by Bispecific CrossMab in Combination with Chemotherapy

**Figure 2:**
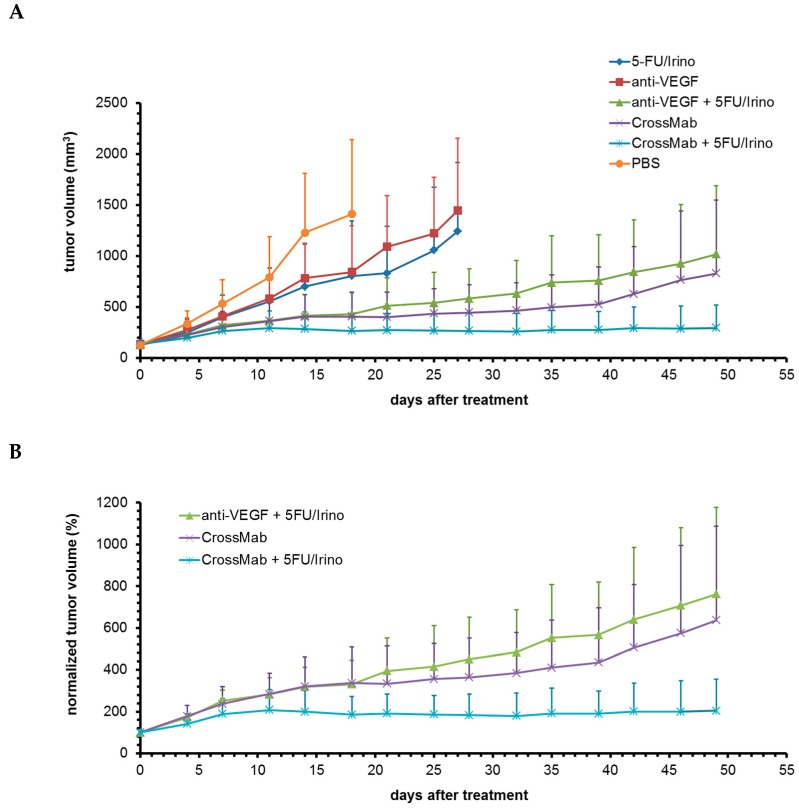
Analysis of tumor growth inhibition comparing CrossMab mediated co-targeting Vascular endothelial growth factor (VEGF) and Ang-2 vs. clinical standard anti-VEGF treatment in combination with chemotherapy. Mice were treated once weekly starting on day 0. (**A**) All groups. Values are means of tumor volumes ± standard deviation (*n* = 10). (**B**) Three most responsive groups. Shown are mean tumor volumes normalized to day 0 as percent ± standard deviation (*n* = 10).

To investigate the impact of co-targeting VEGF and Ang-2 in combination with chemotherapy, a larger trial using 10 mice per group was conducted and efficacy of the bispecific CrossMab were compared to clinical standard anti-VEGF- and chemotherapy in accordance with a FOLFIRI + bevacizumab regimen. Each group consisted of equal distributed tumor volumes and similar mean volumes ± standard deviation at start of treatment with 132 + 56 mm^3^, 134 + 44 mm^3^, 128 + 45 mm^3^, 131 + 55 mm^3^, 137 + 42 mm^3^, 127 + 33 mm^3^ for 5-FU/Irino, anti-VEGF, anti-VEGF+5-FU/Irino, CrossMab, CrossMab+5-FU/Irino, PBS control, respectively. Results of the trial are summarized in Figure 2A. The depicted standard deviations demonstrate the typically observed heterogeneity of the model which results from a variable increase of tumor volumes independent of the start volumes during normal growth (PBS control group) but also under treatment which was already observed in the preliminary study. The control group reached a mean tumor volume of 1400 mm^3^ on d18 after start of treatment with 4 of 10 tumors exceeded 1500 mm^3^ and treatment was stopped. Chemotherapy and anti-VEGF treatment inhibited tumor growth to some extent and had to be completed on d27 when four tumors within each group exceeded 1500 mm^3^. This confirmed the limited impact of combination chemotherapy in the DLD1 xenograft tumor model. The anti-VEGF treatment induced; specific transient growth retardation phase observed in the preliminary study was lesser pronounced in this trial. More efficient tumor growth inhibition could be achieved by single treatment with the CrossMab and with the combinations of either anti-VEGF or CrossMab with chemotherapy allowing further treatment. The study was completed on d49 when two tumors within the CrossMab group and two tumors within the anti-VEGF/chemotherapy group had exceeded 1500 mm^3^. Mean tumor volumes at this time point were 1018 mm^3^, 829 mm^3^ and 296 mm^3^ for anti-VEGF/chemotherapy, CrossMab and CrossMab/chemotherapy, respectively. 

**Figure 3:**
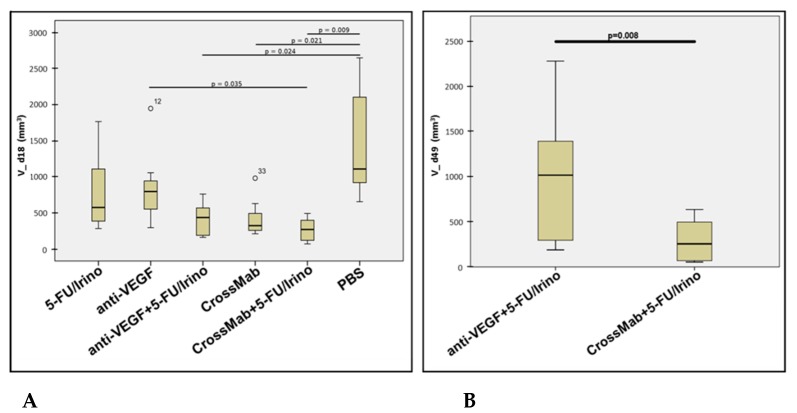
Statistical analysis of tumor volumes (**A**) on day 18 (V_d18) comparing all groups (ANOVA, Post Hoc Dunett-T3) and (**B**) comparing both combination therapy groups on day 49 (V_d49) after completion of study (Student’s T-Test).

Next, for a precise comparison of specific tumor response among the 3 groups with the most active therapy regimens, the increase of tumor volumes was analyzed dependent on the initial volume at start of treatment and depicted as percent. This resulted in a similar response pattern but separated the CrossMab/chemotherapy group more clearly from the two other groups (Figure 2B). Considering of single tumors within each group revealed that 3 of 10 tumors in the anti-VEGF/chemotherapy group, 6 out of 10 tumors in the CrossMab group and 10 out of 10 tumors in the CrossMab/chemotherapy group had shown complete growth retardations with tumor stasis in different volume stages until completion of the study. The other seven tumors within the anti-VEGF/chemotherapy group showed a slow but continuous and variable growth during the course of treatment. In the CrossMab group one tumor initially grew and retarded on d18 to continue growth from d32 on. The other three tumors began to grow on day 21, 39, 42, respectively. The variable tumor response within both groups is clearly represented by the increasing standard deviations and is in contrast to the pattern of the CrossMab/chemotherapy group where complete retardation and stasis in all tumors have led to narrow tumor volume distribution (Figure 2B) which could also be recapitulated after statistical analysis (Figure 3).

A statistical analysis was performed considering values on d18 (completion of control group) comparing all groups. This revealed that treatment with CrossMab alone and both combination treatment regimens have led to a significant inhibition of tumor growth compared to PBS control, with CrossMab/chemotherapy treatment as the most significant (Figure 3A). The latter was also significant superior to anti-VEGF mono treatment. Both combination treatment regimens were compared on day 49 at the end of the study. This demonstrated a highly significant difference in favor of the CrossMab containing regimen (Figure 3B). 

**Figure 4:**
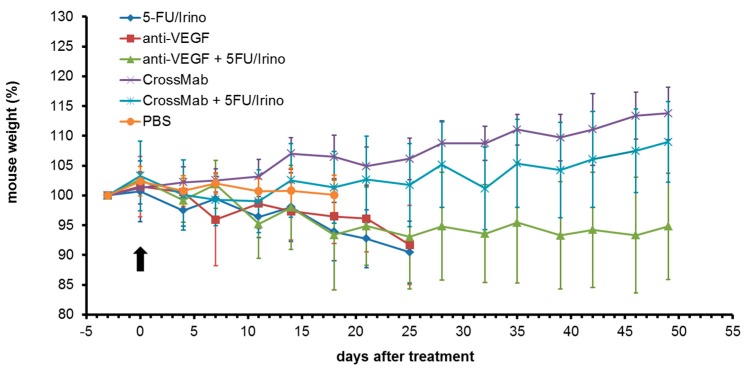
Analysis of mouse weight during treatment normalized to pre-treatment weight. Mice were treated once weekly starting on day 0 (arrow). Values are means ± standard deviation (*n* = 10).

Lastly, mouse welfare with regard to treatment regimens were evaluated analyzing behavior and weight of mice. As shown in Figure 4, CrossMab alone and CrossMab/chemotherapy treatment were very well tolerated by mice resulting in weight gain. Chemotherapy alone and anti-VEGF containing treatment led to weight loss but to a tolerable extent. No alterations in mouse behavior were observed.

### 2.3. Histological Analysis of tumors

**Figure 5:**
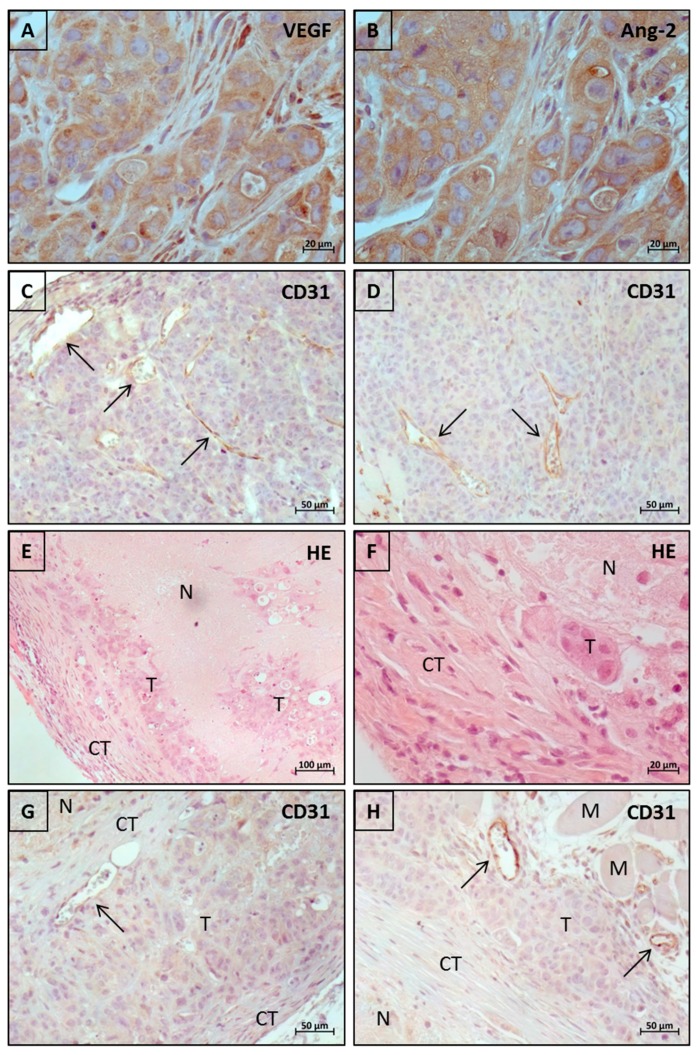
Histological examination of tumor tissues. (**A**,**B**) Staining of VEGF and Ang-2 shows cytoplasmic localization in tumor cells and stromal cells (magnification 400×). (**C**) CD31-positive vessel structures with typical pattern throughout vital tumor tissue and at the periphery (upper left) from a tumor of control group (magnification 200×). (**D**) Normal CD31-positive vessel formation within vital tumor tissue in a CrossMab treated, initially retarded and later progressive tumor (magnification 200×). (**E**) Representative example of a complete retarded tumor showing massive intratumoral necrosis surrounded by layers of vital tumor cells and connective tissue with avascular appearing tumor tissue islands (magnification 100×). (**F**) Peripheral area of the smallest tumor residue from the CrossMab/chemotherapy group showing a small tumor cell colony embedded in connective tissue (magnification 400×). (**G**) Peripheral area of a tumor residue from the CrossMab/chemotherapy group showing CD31-positive vessel structure located at the border between tumor tissue and an inner layer of connective tissue that separates the central necrotic area from the tumor tissue; the outer layer of connective tissue is seen lower right (magnification 200×). (**H**) Peripheral area of a tumor residue from the CrossMab/chemotherapy group showing CD31-positive vessels between muscle tissue from the mouse body and invading tumor tissue outside of the outer connective tissue layer of main tumor area. Note the colonizing tumor cells around marked vessels (magnification 200×). (N—necrotic area, T—tumor tissue, CT—connective tissue, M—muscle tissue; arrows points to CD31-positive vessel structures).

For histological examination of resected tumors, HE-staining and immunohistochemistry for VEGF, Ang-2 and CD31 were performed. Tumor tissue showed strong expression of both VEGF and Ang-2 (Figure 5A,B) which was in accordance with the observed impact of targeting each single factor but also with the improvement mediated by co-targeting both factors as shown in Figure 1. Different tumor responses were associated with different histological features. Tumors that did grow under therapy, e.g., in anti-VEGF and chemotherapy groups, were characterized by typical CD31-positive vessel structures within vital tumor tissue and at the periphery as shown in Figure 5C. Such structures could also be observed in those 7 tumors of the anti-VEGF/chemotherapy group that showed slow growing. Notably, the single CrossMab treated and initially retarded tumors that later became progressive under therapy, apparently showed normal vessel formation within vital tumor tissue although to a lesser extent (Figure 5D). Tumors that had shown complete growth retardations were characterized by massive intratumoral necrosis surrounded by layers of vital tumor cells and connective tissue with avascular appearing tumor tissue islands (Figure 5E). In smaller tumor residues, which preferentially could be obtained from the CrossMab/chemotherapy group, the amount of residual tumor tissue was reduced, respectively. However, even in the smallest tumor residue, which appeared tumor free, few residual tumor cells could be found after thorough examination (Figure 5F). Typically, complete retarded tumors showed CD31-positive vessel structures restricted to the periphery where they were located at the border between tumor and connective tissue but belonging to the main tumor area (Figure 5G). In some cases, CD31-positive vessels were located between muscle tissue from the mouse body and invading tumor tissue outside of the main tumor area where the vessels obviously belong to the muscle tissue (Figure 5H). 

## 3. Discussion

In the present study we evaluated the efficacy of co-targeting VEGF and Ang-2 in combination with chemotherapy in a colorectal carcinoma model. Combined inhibition of VEGF and Ang-2 had been supposed to improve anti-angiogenic therapy. With vanucizumab, a novel bispecific monoclonal antibody that binds both VEGF and Ang-2, the new approach of co-targeting both factors was recently tested in a clinical phase I study [17]. Interestingly, encouraging antitumor activity was observed in one patient with metastatic colorectal cancer. The bispecific CrossMab used in this study represents the murine counterpart of vanucizumab optimized for preclinical investigations (see material and methods). It recognizes both human and murine targets. Therefore, the in vivo condition in mouse xenograft studies when using the CrossMab is in accordance with those in patients. This holds true for both antibodies used to target single VEGF and Ang-2. 

In our first trial we compared single targeting vs. co-targeting VEGF and Ang-2. Overall, an impact of targeting each single factor but also a clear advantage of co-targeting both factors could be demonstrated. Furthermore, Ang-2 was confirmed, in addition to VEGF, as another important factor for tumor angiogenesis in the used colorectal cancer model. The high tumor inhibitory activity of CrossMab mediated co-targeting of VEGF and Ang-2 as well as the clear superiority over single VEGF targeting could be reproduced in our second trial. In addition, the relevance of both factors in the used model was further confirmed by immunohistochemical analyses since the tumor tissue showed strong staining for both VEGF and Ang-2 (Figure 5A,B). This was a prerequisite to evaluate the impact of co-targeting both factors in combination with chemotherapy compared to the clinical standard regimen of anti-VEGF/chemotherapy where Ang-2 is not addressed yet. 

In clinical practice, targeted drugs will eventually be combined with conventional chemotherapy after first testing to further improve therapeutic efficacy. However, resistance to chemotherapy is another present problem in the clinic. Therefore, one aim of our study was to evaluate the impact of chemotherapy on anti-angiogenic therapy in the context of chemotherapy resistance. For this purpose, we used xenografts derived from the colorectal carcinoma cell line DLD1 which is resistant to various drugs including 5-FU and irinotecan. Accordingly, treatment with chemotherapy comprising both drugs only resulted in limited tumor growth inhibition in this model. Nevertheless, a clearly improved therapeutic efficacy could be observed when chemotherapy was added to anti-VEGF treatment which alone was also lesser effective. This demonstrates the beneficial effect of combining chemotherapy with anti-angiogenic therapy in the context of chemotherapy resistance. Moreover, even the high effective CrossMab mediated anti-angiogenic treatment benefited from combination with chemotherapy since addition of chemotherapy obviously prevented tumor regrow that can occur under CrossMab single treatment. Finally, comparison of both combination treatment regimens demonstrated a highly significant difference in favor of the CrossMab containing regimen. 

Notably, the effect obtained by CrossMab single treatment outperformed that of anti-VEGF/chemotherapy combination treatment, although the graph of the CrossMab group shown in Figure 2A seems to eventually converge with the graph of the anti-VEGF/chemotherapy group. This can be explained by the fact that those tumors within the CrossMab group which began to grow later then grew faster thereby more influencing the mean tumor volume. Interestingly, well formed CD31-positive vessels within vital tumor tissue could be found in these tumors (Figure 5D). Also, the tumor tissue appeared to be lesser compact as compared to control tumors (Figure 5C,D). The observed tumor growth combined with normal vessel formation despite further treatment points to other existing or evolving angiogenic mechanisms in addition to or complementary to VEGF and Ang-2. Indeed various mechanisms based on alternative angiogenic factors such as FGF, PDGF, PlGF, Bv8, IL-1 or TGF-beta have been described which could be relevant in this context [18]. The colorectal carcinoma xenograft model used in this study offers an ideal model to investigate the mechanisms responsible for resistance to anti-angiogenic therapy mediated by co-targeting VEGF and Ang-2 since some tumors will start growing under therapy in a reliable manner and the onset can be monitored easily, respectively. On the other hand, the well-formed vessels found in CrossMab refractory tumors could also point to another effect achieved by anti-angiogenic therapy which is called vessel normalization. This is in contrast to the classical view of the impact of anti-angiogenic therapy which aims at inducing vascular regression followed by deprivation of oxygen and nutrients to stop tumor growth. Interestingly, a vascular normalization effect was reported in the first preclinical study using this antibody [16]. Moreover, tumor vessel normalization and thereby improving drug delivery has been suggested as an alternative concept employing anti-angiogenic therapy [19], although further research has revealed that this could be restricted to small molecules such as chemotherapeutic agents [20]. Based on these notions, it can be speculated that those tumors within the CrossMab group which began to grow would not do so if co-treated with chemotherapy. This is supported by the fact that combination treatment comprising CrossMab and chemotherapy achieved complete and durable tumor growth retardation in 10 of 10 cases. However, none of these tumor residues were completely free of tumor cells which suggest that tumors will eventually relapse after cessation of therapy. Nevertheless, the CrossMab/chemotherapy combination regimen achieved an absolute tumor growth control for a long time and was clearly superior compared to the clinical standard anti-VEGF/chemotherapy regimen. Notably, it was very well tolerated. 

Complete tumor retardation was associated with histological features as represented in Figure 5E with massive intratumoral necrosis surrounded by layers of vital tumor cells and connective tissue. As shown in Figure 5G, CD31-positive vessel structures were restricted to the periphery and appeared to be well-formed indicating occurrence of vessel normalization. Together these observations suggest that the underlying mechanism of the complete and durable tumor retardation achieved by the CrossMab/chemotherapy regimen is a result of two combined effects, namely strong vascular regression followed by massive necrosis but also normalization of remaining vessels leading to facilitated delivery of chemotherapy and inhibition of tumor cell residues. A similar observation was made in a recent study using the CrossMab where these effects have led to facilitated accumulation of immune cells [21].

In some cases, CD31-positive vessels were located between tumor tissue and adjacent muscle tissue from the mouse body outside of the main tumor area indicating tumor invasion (Figure 5H). These vessels obviously belong to the muscle tissue. Interestingly, some tumor cells directly colonize these vessels and the tumor tissue seem to be poised to grow around the vessels (Figure 5H). This behaviour points to a mechanism called vessel co-option where tumor vascularization can occur non-angiogenic and tumor cells instead incorporate pre-existing vessels from surrounding tissue and which have several tumor biological consequences with respect to metastatic growth and in particular for anti-angiogenic therapy [22]. Notably, such vessels are not vulnerable to anti-angiogenic therapy. Indeed, vessel co-option has been described as mechanism mediating resistance to bevacizumab and sunitinib based anti-angiogenic therapy [23,24]. Together, this suggests that anti-angiogenic therapy mediated by co-targeting VEGF and Ang-2 could eventually be affected by the same resistance mechanism as bevacizumab at least if caused by vessel co-option. Moreover, the necrotic and hypoxic microenvironment in complete retarded tumor residues could trigger the selection of more resistant and invasive tumor cells which then can spread via vessel co-option. Therefore, a possible treatment related effect on tumor metastasis should be investigated in further studies. 

In summary, the data from this model clearly support the strategy of co-targeting VEGF and Ang-2 and further demonstrate the beneficial impact of co-treatment with chemotherapy. In addition, the used model is applicable to further investigate angiogenic mechanism that can occur despite co-inhibition of VEGF and Ang-2 and to test new additional therapeutic treatments targeting vessel co-option. The clear superiority of the CrossMab-containing regimen compared to clinical standard anti-VEGF/chemotherapy warrants further analyses in other models.

## 4. Materials and Methods 

### 4.1. Antibodies

The following therapeutic antibodies which were kindly provided by Roche Diagnostics GmbH (Penzberg, Germany) were used:

(1) anti-VEGF: chimeric mouse IgG2a mAb based on mAb B20-4.1 (RO6872895) [25]
(2) anti-Ang-2: chimeric mouse IgG2a mAb based on mAb LC06 (RO6872894) [26]
(3) anti-Ang-2-VEGF bispecific CrossMab: chimeric mouse IgG2a based on LC06 and B20-4.1 (RO6872840, murinized A2V) [21,27]

All antibodies recognize both human and murine targets, respectively. Solutions of antibodies for application were prepared in normal PBS.

### 4.2. Animal Studies 

The investigations of this study were approved by the Laboratory Animal Care Committee of Sachsen-Anhalt, Germany, and were performed according local guidelines. Subcutaneous xenograft tumors were generated in athymic nude mice (Charles River Laboratories) using the human colorectal cancer cell line DLD-1. Five million cells were inoculated into the right flank of male athymic nude mice. Monitoring of tumor growth was performed by caliper measurement and volume calculation using the formula a^2^ × b × π/6 with ‘**a**’ being the short and ‘**b**’ the long dimension. Treatment was performed once weekly by intraperitoneal injections. Chemotherapy consisted of a combination of 5-fluorouracil (30mg/kg bodyweight) and irinotecan (50mg/kg). The therapeutic antibodies were administered at a dose of 10mg/kg alone or in combination with 5FU/Irino chemotherapy. Control mice received PBS. Mouse weight and behavior were controlled daily during the course of treatment. 

### 4.3. Histological Analysis of Tumors 

Necropsied tumors were cross-sectioned, fixed in 5% formalin and embedded in paraffin according standard protocols. For hematoxylin and eosin (HE) staining and immunohistochemical analysis, 4–5 μm slices were prepared with a RM 2245 microtome (Leica, Wetzlar, Germany). Slices were dewaxed and rehydrated by decreasing alcohol series from xylene up to bi-distilled water. HE staining was performed according standard protocols using hematoxylin (Dako, Hamburg, Germany) and eosin (Merck Chemicals GmbH, Darmstadt, Germany). For immunohistochemical analysis, slices were pre-treated 30 min with Target-Retrieval-Solution (Dako, Hamburg, Germany) followed by treatment with Peroxidase-Blocking-Reagent for 20 min (Dako, Hamburg, Germany). Protein blocking was performed with 3% BSA/PBS solution for 20 min. Primary antibodies prepared in 1% BSA/PBS were incubated for 3 h at RT followed by washing with PBS. Biotin-conjugated secondary antibodies prepared in PBS were incubated for 1h followed by washing and incubation with Streptavidin/HRP-complex (Dako, Hamburg, Germany). After washing, slices were incubated with DAB+ Substrate Chromogen System (Dako, Hamburg, Germany). The slices were dehydrated by ascending alcohol series and fixed with Roti^®^-Histokit (Carl Roth GmbH & Co. KG, Karlsruhe, Germany). Following antibodies were used: rabbit polyclonal anti-VEGF (A20), sc-152 (Santa Cruz Biotechnology); goat polyclonal anti-Ang-2 (AF623) (R & D Systems); anti-CD31, ab28364 (Abcam); anti-goat-IgG-B, anti-rabbit-IgG-B (Santa Cruz Biotechnology).

### 4.4. Statistical Analysis

All calculations and the generation of the box plots were performed with SPSS (IBM Cooperation). Groups were treated as normally distributed. The tumor volume was set as dependent variable. The influencing factor was the treatment. Significant differences between treatments were analyzed using the Levene Test (variance homogeneity) prior to Oneway ANOVA (group differences) and Post Hoc Dunnett-T3 (identification of significant group differences) on study day 18 (for all groups (*n* = 10)). After completion of the study (day 49), the two-combination treatment regimen (different antibodies plus chemotherapy (*n* = 10)) were compared using Student’s T-Test. Significance level was always set to α = 0.05.
